# Prevalence of cystic echinococcosis in slaughtered livestock in Iran: a systematic review and meta-analysis

**DOI:** 10.1186/s12879-021-06127-2

**Published:** 2021-05-07

**Authors:** Aliakbar Vaisi-Raygani, Masoud Mohammadi, Rostam Jalali, Nader Salari, Melika Hosseinian-Far

**Affiliations:** 1 Department of Nursing, School of Nursing and Midwifery, Kermanshah University of Medical Sciences, Kermanshah, Iran; 2 Department of Biostatistics, School of Health, Kermanshah University of Medical Sciences, Kermanshah, Iran; 3 Department of Food Science & Technology, Faculty of Agriculture, Ferdowsi University of Mashhad (FUM), Mashhad, Iran

**Keywords:** Hydatid cyst, *Echinococcus granulosus*, Livestock, Cystic echinococcosis, Meta-analysis

## Abstract

**Background:**

Hydatidosis is a zoonotic disease and has a great general and economic health importance in both developed and developing countries. Therefore, this systematic and meta-analytic study was conducted to determine the prevalence of cystic echinococcosis in slaughtered livestock in Iran.

**Methods:**

The present study was conducted as a systematic review and meta-analysis. The SID & Magiran, MEDLINE (PubMed), ScienceDirect, Scopus, and Google Scholar databases were searched with a view to selecting relevant research works. As a result, 31 articles published from April 1970 to April 2020 were selected. The heterogeneity of the studies was assessed using the I^2^ index. Data analysis was conducted within the Comprehensive Meta-Analysis software (CMA) v.3.0 (Biostat, Englewood, NJ, USA) and Arc map (ArcGIS 10.3) software.

**Results:**

The heterogeneity of the studies was evaluated using the I^2^ test which value was 99% showing a high heterogeneity in the studies. The results of publication bias in studies were evaluated by the Egger test, which were not statistically significant (*P* = 0.144). The overall prevalence of cystic echinococcosis in slaughtered livestock in Iran is 13.9% (95%CI: 10.7–17.7%). The results of the meta-regression analysis indicate the increasing trend of the hydatid cyst prevalence with the increase of sample size and publication year (*P* < 0.05).

**Conclusion:**

According to the results of this study and the relatively high prevalence of cystic echinococcosis in slaughtered livestock in Iran, health policy makers should make effective decisions in this regard, and implement careful inspections and interventions by experts and health authorities.

## Background

Hydatid cyst is the larval stage of *Echinococcus granulosus*, a 3-7 mm worm in dog’s intestine, where the worm’s eggs are dispersed in the environment by the infected dog’s stool. *E. granulosus* is a cyclophyllid cestode and belongs to the Echinococcus genus; it includes 10 main genotypes (G1-G10), Sheep strain (G1), Tasmanian sheep strain (G2), Buffalo strain (G3), Horse strain (G4), Cattle strain (G5), Camel strain (G6), Pig strain (G7) and Cervid strain (G8), human polish strain (G9), and Fennoscanadian cervid strains (G10) [1, 2].

In the evolutionary cycle of this parasite, wild and domestic carnivores especially dogs are the final host, with herbivores being the intermediate hosts of this parasite and humans are accidental intermediate hosts [2]. Livestock are infected by eating these eggs through water, food, and vegetables, after which the hydatid cysts form in their bodies [3].

Although the infection of carnivores with the mature stage of the worm does not cause a particular problem, the establishment of larvae (cyst) in various organs, especially the liver and lungs, and sometimes brain, heart and spinal cord of the intermediate host, like humans, cause hydatidosis, However, rupture of a cyst results in trauma and physical internal injury, and can also cause more severe complications [3].

This, in turn, causes its components to reach other tissues through bloodstream, causing severe and even fatal diseases [4, 5]. The clinical symptoms of hydatidosis in humans and livestock depend on the number, size, and location of the formed cysts. The importance of the disease in humans is due to the involvement of vital organs such as the liver, lungs, while in domestic livestock and cattle, it is due to the significant economic loss [6, 7]. Given the considerable economic losses due to hydatidosis in the public health and livestock sector, this emerging and re-emerging disease is considered as one of the major health and economic concerns [8].

Hydatidosis has a worldwide distribution and is endemic in some parts of the world such as Australia, North Africa, and the Middle East. It is also reported to be widespread in most parts of Iran [9–11]. Stray dogs and herds are key disseminators of the infection across Iran, nevertheless, wild carnivores such as yellow jackals and red foxes also maintain the parasite life cycle in some parts of the Country [12].

The rate of animal contamination in the Country has been reported to be between 1.5 and 64% in sheep, goat, cattle, buffalo, and camel. Due to the difficulty in diagnosis and treatment of hydatid cyst and the risks of this disease for humans, disease control and prevention are vital throughout the world [12, 13].

Moreover, due to the zoonotic nature of the disease, as well as its health, medical, and economic importance, conducting a study on the prevalence of disease in livestock populations and having an effective prevention and control plan for the disease is required [14]. Furthermore, the overall prevalence of cystic echinococcosis in slaughtered livestock in Iran is still unknown. Accordingly, this piece of research intends to answer the following research question: ‘what is the overall Prevalence of cystic echinococcosis in slaughtered livestock in Iran?’ Since there are inconsistent reports on the prevalence of the disease in different regions of Iran, this study aimed to conduct a systematic review and a meta-analysis to overall the prevalence of cystic echinococcosis in slaughtered livestock in Iran.

## Methods

This study was conducted in accordance with the criteria of the Preferred Reporting Items for Systematic Reviews and Meta-Analyzes (PRISMA) and Cochrane seven-step approach. Based on which, selection of research questions, systematic search of databases, organization of documents for review, selection of studies in accordance with the criteria defined by the authors, information extraction, analysis and finally the presentation of the final report were implemented.

### Research question and determining the keywords

Systematic search of articles was performed in Iranian databases including (SID, Magiran) as well as the international databases of Google scholar, MEDLINE (PubMed), Scopus, ScienceDirect.

The keywords used for the search in this study were selected based on published preliminary studies and also Medical Subject Headings (MESH Terms) in the reviewed database. Also, a detailed study of the questions in this study and the keywords were selected according to PECO criteria.

PECO criteria included: Participants: In this study, total livestock studied in Iran, Exposure: cystic echinococcosis, Comparison: cystic echinococcosis was considered in the total livestock studied in Iran, Outcomes: The overall prevalence of cystic echinococcosis in slaughtered livestock in Iran was reported by Species of livestock and Regions of Iran and sample size. The search process in Persian databases was done using Persian keywords, and English equivalent words were used in the English databases including livestock, slaughterhouse, hydatid cyst, Echinococcosis, cystic echinococcosis. Also, in This study the AND/OR operators, were used to provide more comprehensive access to all articles. Therefore, the AND/OR operator was used to check the common names for the disorder like by matching words in the MeSH browser. The search was conducted in various databases April 1970 to April 2020. References to past related studies and the Google Scholar search engine were also further explored to find relevant empirical studies.

### Inclusion and exclusion criteria

Inclusion criteria included cross-sectional studies that focused on the prevalence of cystic echinococcosis in slaughtered livestock in Iran, studies that have the full text available and the information in the present study, including the study sample and the number of slaughtered livestock with cystic echinococcosis and exclusion criteria included observational studies such as control case and cohort studies, case report studies, case series, review studies, intervention and clinical trial studies.

### Selection of studies

After collecting the studies researched in EndNote reference management software version X7, for Windows, Thomson Reuters), the studies were started by the authors. Evaluations in this study were performed independently and blinded. Initially, two researchers (NS and AVR) reviewed the titles and abstracts of articles. In case of disagreement among the researchers regarding each of the articles, the third party (MM) reviewed and provided the final opinion regarding that study. Then, the full text of the studies confirmed in the initial evaluation was reviewed by the same researchers in terms of criteria defined according to the PECO criterion.

### Qualitative evaluation of studies

The quality of confirmatory studies in the previous stages was measured by the methodological quality assessment tool of observational studies. The STROBE checklist was used in this study. This checklist examines various aspects of writing a study, including title, study objectives, study type, population, sample size, study data collection tools, statistical analysis. A score was assigned in the range of 0–32 to the studies. Due to the fact that in this systematic review, studies with good or average quality were included in the analysis, articles that received a score of 12 and above were selected by the authors, and studies with a score of less than 12 were considered to be of poor quality and excluded.

### Statistical analysis

Data was extracted through pre-designed forms. Various criteria such as demographic information (first author, year of publication, Kind of animal checked, Area, Sample size and prevalence), were extracted and entered into the relevant forms and Comprehensive Meta-analysis (Biostat, Englewood, NJ, USA version 3) was used to analyze the data. The Egger test and the corresponding Funnel plot were used to investigate the publication bias. The I^2^ index was used to assess the heterogeneity of the selected research works.

### Geographical study of the prevalence of cystic echinococcosis

For this purpose, the information extracted from the meta-analysis was entered into Arc map software (ArcGIS 10.3) software and the cystic echinococcosis prevalence was reported using maps drawn by the software.

## Results

### Search output

**Fig. 1:**
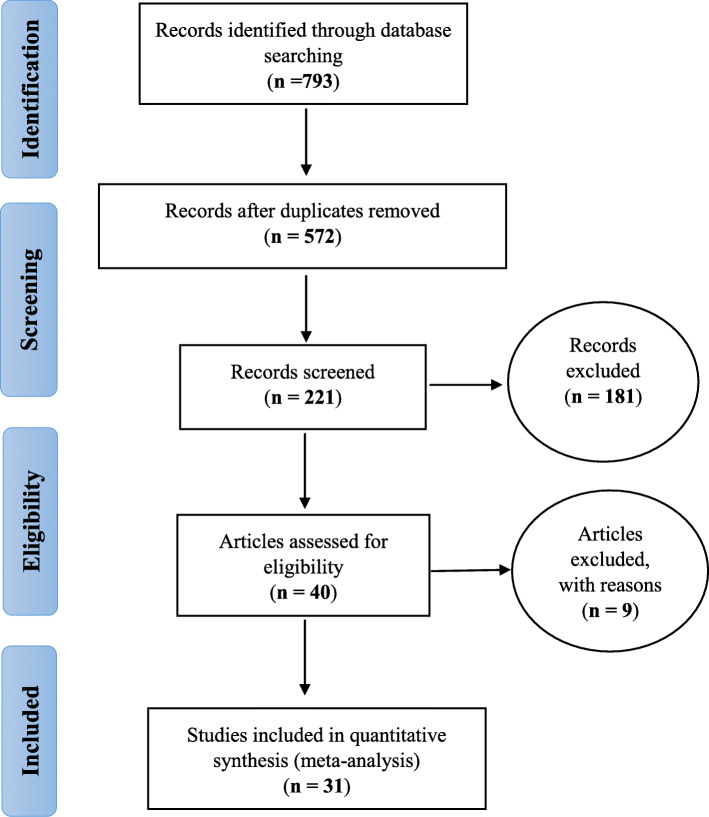
The PRISMA flow diagram for this systematic review and meta-analysis

In the present study, all studies performed on the prevalence of cystic echinococcosis among slaughtered livestock in Iran were examined systematically based on the PRISMA guidelines. In the initial search, 724 studies were identified, from which 31 studies published between April 1970 to April 2020 entered the final analysis [15–45] (Fig. 1).

Data of from all the final studies were extracted using a different pre-prepared checklist. The items on the checklist included: author’s name, article title, year and location of the study, the domestic animal studied, sample size, and the prevalence of cystic echinococcosis among the slaughtered livestock in Iran [15–45] (Table 1).

**Table 1:** Specifications of the studies included in this work

Row	Author [References]	Published	Area	Kind of animal checked	Sample size	prevalence
1	Fallah [15]	2008	Hamedan and Brujerd	Sheep, Cow, Goat	5709	13.5
2	Delimi asl [16]	2001	Bushehr	Sheep, Cow, Goat	67,840	5.3
3	Adeli-Sardooei [17]	2015	Kerman	Sheep	11,580	5.1
4	Esmaeilzadeh [18]	2013	Ahwaz	Sheep	4592	0.7
5	Khanjari [19]	2010	Tehran	Sheep, Cow	567,559	10.03
6	Hamzavi [20]	2016	Hamedan (Asadabad)	Sheep, Cow, Goat	12,000	10.7
7	Sadeghi [21]	2012	Kurdestan (Baneh)	Cow	400	67.7
8	Azami [22]	2013	Isfahan	Sheep, Cow, Goat	196,325	9.3
9	Aminpour [23]	2012	Urmieh, Tabriz,Ardebil,Gilan, Ahwaz	Buffalo	3832	9
10	Borji [24]	2012	Mashhad	Sheep, Cow, Goat	5,131,485	29.7
11	Daryani [25]	2007	Ardebil	Sheep, Cow, Goat, Buffalo	5381	60.8
12	Fakhar [26]	2007	Qom	Sheep, Cow, Goat	3400	8.5
13	Mirzaei [27]	2015	Tabriz	Sheep, Cow, Goat, Buffalo	14,828	25.6
14	Mirzaei [28]	2016	Tabriz	Camel	198	14.6
15	Motakef [29]	1976	Khorasan	Sheep, Cow, Goat, Camel	15,691	8.02
16	Mansoorlakooraj [30]	2011	Gilan, Mazandaran, Golestan	Sheep, Cow, Goat	3,347,797	12.7
17	Mehrabani [31]	1999	Shiraz	Sheep, Cow, Goat, Buffalo	6602	5.4
18	Fallah [32]	2014	Hamedan,Brujerd	Sheep, Cow, Goat	5709	13.5
19	Ahmadi [33]	2011	Ahwaz	Sheep, Cow, Goat	3,583,417	4.6
20	Ziaei [34]	2011	Mazandaran	Sheep, Cow, Goat	3119	54.1
21	Oryan [35]	1994	Fars	Sheep	7992	28.3
22	Ezatpour [36]	2015	Lorestan (Delfan)	Sheep, Cow, Goat	6885	18.01
23	Shahbazi [37]	2016	Kermanshah	Sheep, Cow, Goat	663,633	2,.7
24	Nabavi [38]	2014	Sistan and Baluchestan	Cow	3182	13.4
25	Dalimi [39]	2002	West of Iran	Sheep, Cow, Goat, Buffalo	60,047	11.6
26	Mobedi [40]	1970	Tehran	Camel	955	34
27	Sabbaghian [41]	1975	Shahrekurd	Sheep, Goat,	666	6
28	Afshar [42]	1971	south of Iran	Camel	35	42.8
29	Rahimi [43]	2011	Mazandaran	Sheep, Cow, Goat, Buffalo	2,946,551	19.08
30	Moghaddas [44]	2014	Khorasan (North, South, Razavi), Semnan, Yazd, sistan and baluchestan	Camel	438	30.8
31	Ansari-Lari [45]	2005	Shiraz	Sheep, Cow, Goat	844,039	8.2

### Heterogeneity and publication bias

**Fig. 2:**
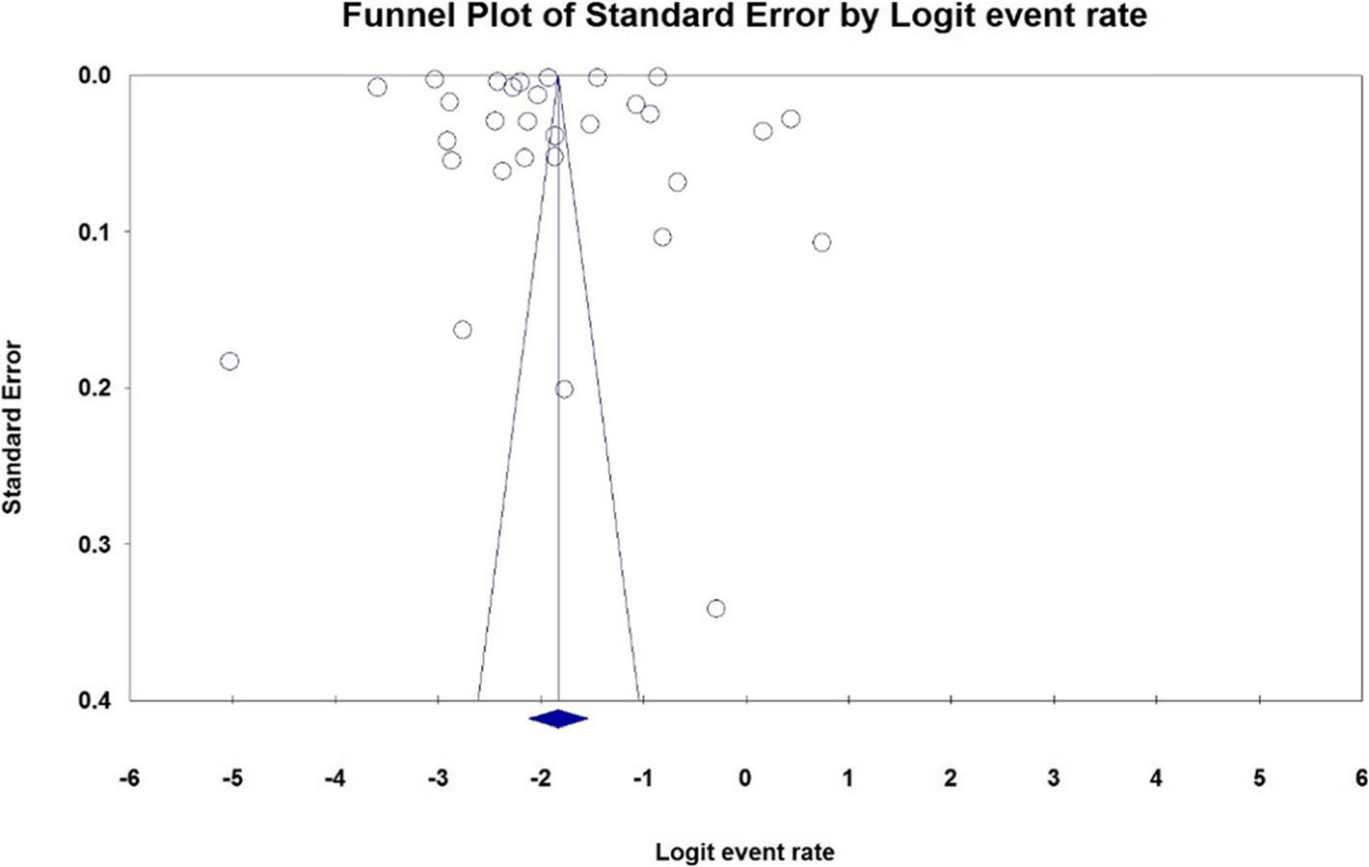
Funnel Plot: The prevalence of cystic echinococcosis in slaughtered livestock in Iran

The results of publication bias in studies were evaluated by the Egger test, which were not statistically significant (*P* = 0.144) (Fig. 2). Also, the heterogeneity of the studies was evaluated using the I^2^ index which value was 99%, Therefore, the random effects model was used to combine the results of the studies.

**Fig. 3:**
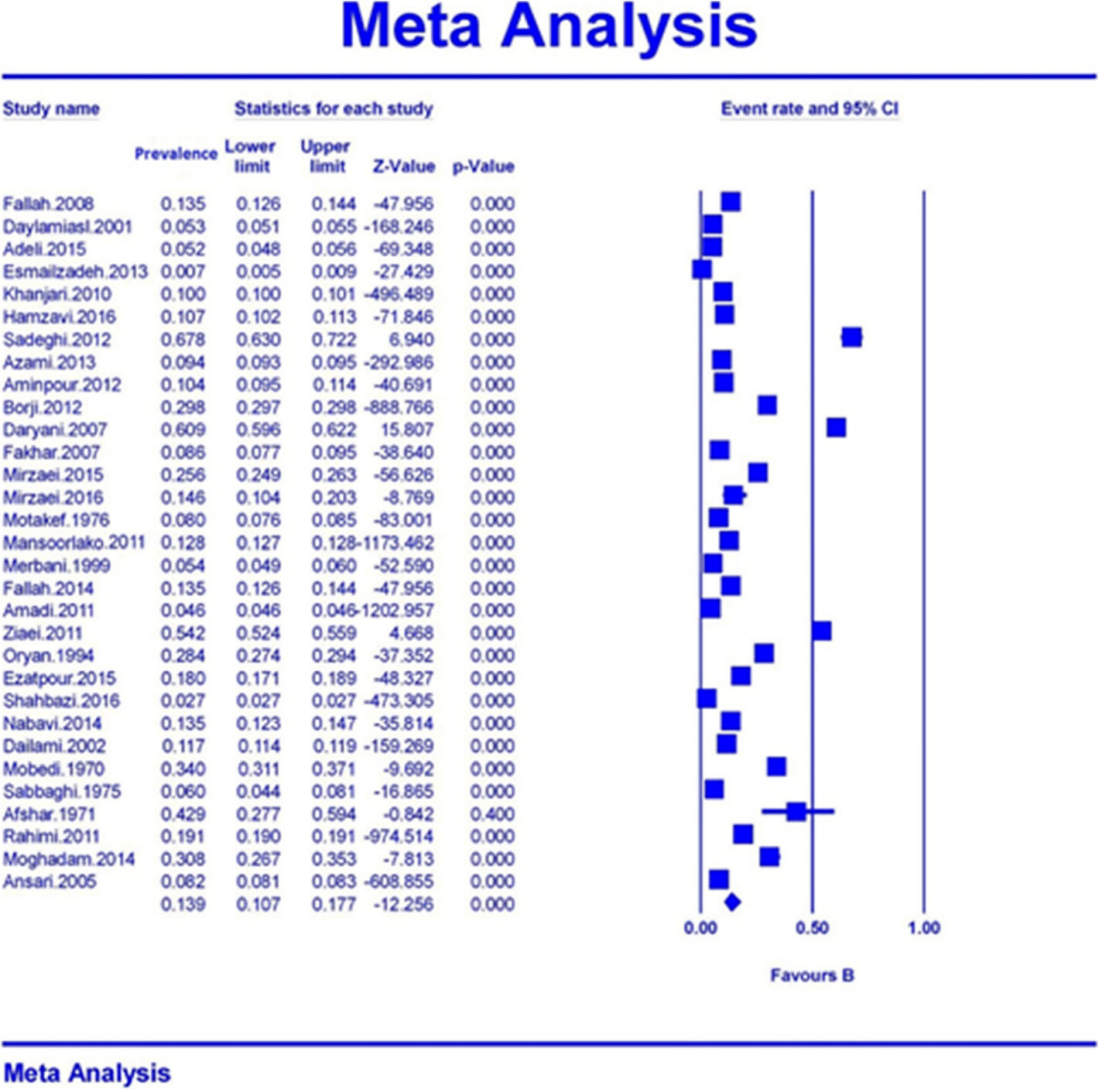
Prevalence of cystic echinococcosis in slaughtered livestock in Iran based on the random model

The highest prevalence of cystic echinococcosis in slaughtered livestock was reported in Baneh with 67.7% (95% CI: 63–72.2) [21], while the lowest cystic echinococcosis in slaughtered livestock was observed in Ahwaz slaughterhouses with 0.7% (95% CI: 0.5–0.9) [18] (Fig. 3). The total number of livestock included in this systematic review and meta-analysis was 17,510,307 consisting of sheep, cattle, goats, buffalos, and camels [15–45]. The overall prevalence of cystic echinococcosis among the slaughtered livestock in Iran based on the random effects model was found to be 13.9% (95% CI: 10.7–17.7).

#### Sub-group analysis

**Fig. 4:**
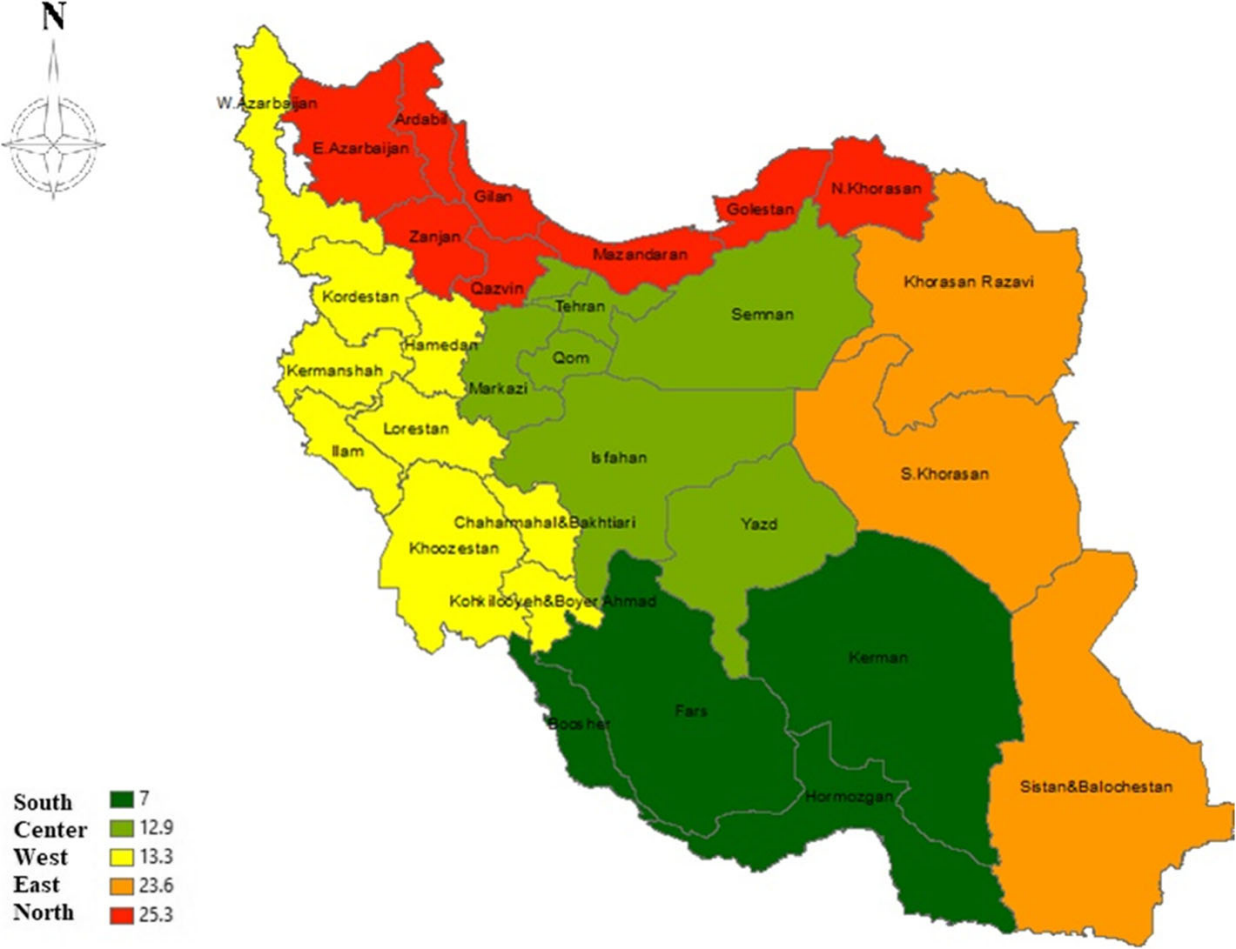
Overall prevalence of cystic echinococcosis in slaughtered livestock in Iran based on the ArcGIS 10.3 (the figure was drawn by the software and was not extracted from another source)

Table 2 presents an analysis of different sub-groups according to Specie of livestock, Regions of Iran, and Sample size (Table 2) prevalence of cystic echinococcosis in slaughtered livestock in Iran were reported based on different geographical areas in Iran and according to the Geographical Information System (GIS) (Fig. 4).

**Table 2:** The results of the sub-group analysis

Variables		No. studies	Prevalence% (95% CI)	I^2^ (%)	*P* value	No. participants
Species of livestock	Sheep	24	4.3 (95% CI: 3.2–5.8)	99.9	0.000	5,694,328
	Goat	20	3.7 (95% CI: 2.6–5.2)	99.9	0.000	5,386,380
	Cow	22	4.8 (95% CI: 3.5–6.5)	99.9	0.000	5,673,408
	Buffalo	6	5.2 (95% CI: 3.5–7.7)	99.1	0.000	762,182
	Camel	5	18.3 (95% CI: 5.5–46.4)	99.3	0.000	5549
Regions of iran	North	7	25.3 (95% CI: 20.4–30.9)	99.9	0.000	6,321,706
	South	9	7 (95% CI: 5.2–9.5)	99.9	0.000	4,541,795
	West	8	13.3 (95% CI: 6.6–24.9)	99.9	0.000	755,049
	East	3	23.6 (95% CI: 13.1–38.7)	99.4	0.001	5,135,105
	Center	4	12.9 (95% CI: 11.3–14.7)	99.4	0.000	768,239
Sample size	< 5000	11	17.9 (95% CI: 9–32.4)	99.6	0.000	20,817
	5000–10,000	6	19.3 (95% CI: 9.3–36)	99.9	0.001	38,278
	10,000–20,000	4	10.6 (95% CI: 4.9–21.6)	99.8	0.000	54,099
	20,000<	10	10.8 (95% CI: 6.7–16.9)	99.9	0.000	17,397,113

#### Meta-regression analysis

**Fig. 5:**
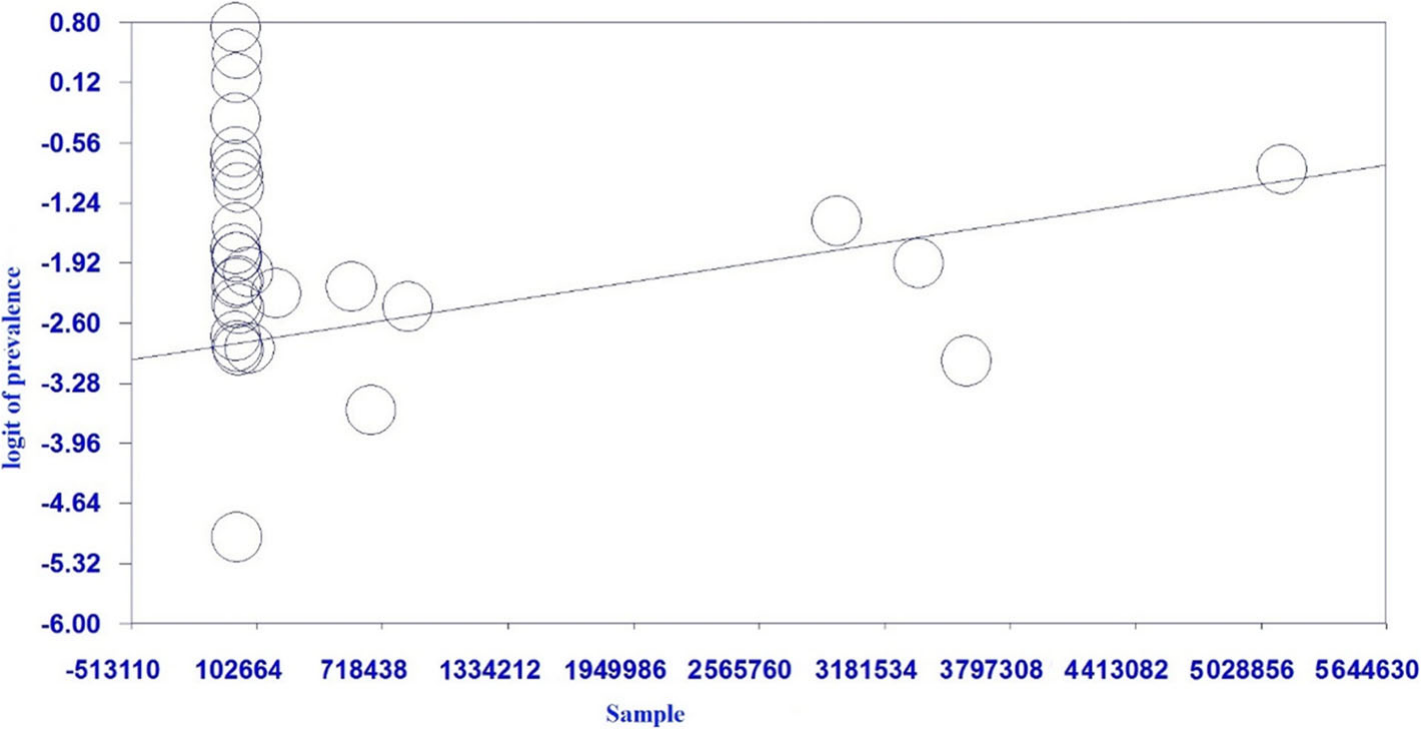
Meta-regression chart showing the frequency of cystic echinococcosis in slaughtered livestock in Iran according to sample size

**Fig. 6:**
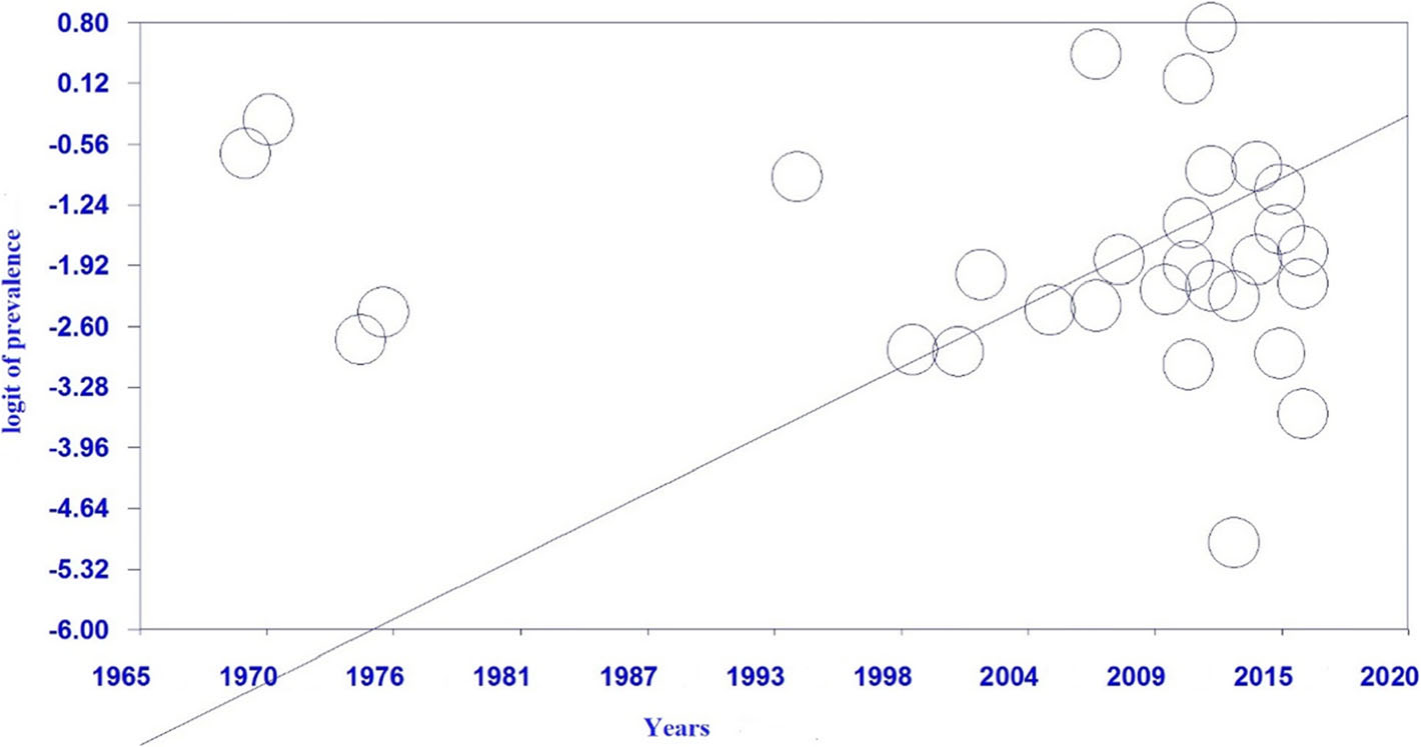
Meta-regression chart representing the frequency of cystic echinococcosis in slaughtered livestock in Iran according to publication year

Accordingly, the results of the meta-regression revealed that any increase in the sample size is associated with a statistically significant growth in cystic echinococcosis prevalence. In other words, studies with larger samples reported significantly higher prevalence of cystic echinococcosis (*P* < 0.05) (Fig. 5). In addition, the increase of the publication year of a study was associated with a significant increase in cystic echinococcosis prevalence, such that the studies published in more recent years had reported significantly higher cystic echinococcosis prevalence compared to the older studies (*P* < 0.05) (Fig. 6).

## Discussion

*E. granulosus* is known as a parasite and veterinary problem in the Middle East. Its intermediate hosts include camels, cows, sheep, and goats and the source for human cystic echinococcosis (CE) is contaminated food and water in which the parasite eggs and livestock are considered as reservoir hosts [46, 47]. The results of our study suggest that the overall prevalence of cystic echinococcosis in slaughtered livestock in Iran is 13.9%.

The prevalence of *E. granulosus* in livestock in slaughterhouses is inconsistent in different countries. This can be due to the difference in the knowledge of the health inspectors, poor carcass inspection facilities in slaughterhouses, regional differences, and research methodologies [48, 49]. The prevalence of hydatid cyst in the livestock in slaughterhouses in Iran is relatively high (13.9%). On the other hand, the prevalence of hydatid disease in the livestock slaughtered in Asia and in particular in Saudi Arabia [50] is reported as 12.6%. Furthermore, a research work conducted in two slaughterhouses in Bursa region in Turkey [51], revealed this prevalence as 3.6%. In Macedonia [52], the prevalence was reported as 19.03%, and the prevalence of *E. granulosus* in livestock in a slaughterhouse in Libya [53] was assessed as 4.9% among sheep, 4.2% in goats, 2.7% across camels, and 15% in cattle. The overall prevalence of hydatid cyst in the livestock was reported % 6.7. In a study of livestock in Oman [54], the prevalence of *E. granulosus* infection was reported as 3.5% and in China the prevalence of hydatid disease was 9.8% in sheep, 8.4% in cattle, 8.6% in camels, and 8.4% in horses with overall prevalence of hydatid cyst in livestock killed reported as 8.9%. It has been stated that the contamination rate varies between different slaughterhouses across different regions. Considering the prevalence of hydatid disease in livestock slaughtered in other countries around the world, in a study on Italian livestock [55], the prevalence was 75%. Moreover, in a research work on Greek livestock [56], the prevalence was reported as 30.2% in sheep, 7.8% in goats, and 42% in buffalos, with the overall prevalence of hydatid cyst akong the slaughtered livestock was reported as 26.6%. In a study on livestock in Oman [57], the prevalence of *E. granulosus* infection was as 3.5, 0.6, 0.07 and 0.03, in camels, cattle, sheep, and goats respectively.

Raising Livestock plays a key role in human nutrition and socioeconomic development. On the other hand, there is a risk of hydatid cyst and human disease, which may ultimately result in costs incurred to the countries’ economy because of the disease [51, 58]. In one research work, the minimum financial loss due to the removal of carcasses and internal organs was $538 [59]. Meanwhile, in another study at a national level, financial losses due to hydatid disease of cattle, sheep, and goats were reported as 32, 54.1, and 2.7 million dollars [60]. This loss for the removal of carcasses and internal organs of livestock infected with hydatid cysts was estimated as high as 1 billion dollars in Saudi Arabia over a three-year period [50, 60].

Programs implemented in different countries to control and eradicate hydatidosis. Such programmes include long-term planning, proper financing by the government, coordinating all relevant organizations and departments, raising public awareness, training the general public on the disease nature, monitoring food hygiene, minimizing contact with dogs, preventing slaughter of livestock in places other than slaughterhouses, constructing well-equipped and sanitary slaughterhouses, accurately inspecting carcasses in slaughterhouses, and eliminating contaminated organs through sanitization and vaccination of sheep with suitable vaccines such as the EG95 [61]. Considering the above-mentioned challenges, all the measures can be important in fighting hydatidosis in livestock, resulting in the reduction of contamination. After implementing a disease control program, it is crucial to continue the actions that maintain the obtained outcomes of the implemented program. Increasing public awareness about the disease and transmission methods, preventing unauthorized slaughter, and controlling stray dogs are among the main control measures. Young livestock are more commonly slaughtered for meat production, since their meat is lighter in color and cooks faster; also, the prevalence of hydatid cyst is higher in older animals [62]. Qingling et al. [55] reported that the hydatid cyst prevalence increases significantly as the animals grow older. In sheep, the rate was reported as 1.9% before 1 year of age, 8.2% in the age of 1–2 years, and 12.3% in 3–4 years old, and reached 17.2% when the animals were 5–6 years old. Considering the livestock age at the time of slaughter can also be considered as an important factor in reducing the rate of infection spread.

### Strengths & limitations

One of the strengths of the present study was obtaining an overall prevalence of cystic echinococcosis in slaughtered livestock in Iran, and according to the best of our knowledge, such a study was conducted for the first time. Moreover, a meta-regression analysis was conducted in this study for the two factors of ‘sample size’ and ‘publication year’. On the other hand, the most important limitation of the study is related to the inaccessibility of the full-text of some retrieved articles and the lack of information required in some of the research works.

## Conclusion

Considering the results of this research, there is relatively high prevalence of hydatid cyst in livestock in slaughterhouses. Moreover, since hydatid cyst is a risk factor for human health, it is necessary that health policy makers make effective decisions in relation to this disease and implement accurate inspections by health experts and authorities.

## Data Availability

Datasets are available through the corresponding author upon reasonable request.

## Abbreviations

SID: Scientific Information Database
GIS: Geographic Information System
STROBE: Strengthening the Reporting of Observational Studies in Epidemiology for cross- sectional Study
PRISMA: Preferred reporting items for Systematic Reviews and Meta-Analysis
